# Fibrin Glue Sealant: An Effective Modality to Mitigate Postoperative Seroma After Modified Radical Mastectomy

**DOI:** 10.7759/cureus.32789

**Published:** 2022-12-21

**Authors:** Shivam Sharma, Jainendra K Arora, Rakesh Kumar

**Affiliations:** 1 General Surgery, Vardhman Mahavir Medical College and Safdarjung Hospital, New Delhi, IND; 2 Cancer Surgery, Vardhman Mahavir Medical College and Safdarjung Hospital, New Delhi, IND

**Keywords:** modified radical mastectomy (mrm), fibrin glue, seroma, drain output, breast carcinoma

## Abstract

Introduction

Modified radical mastectomy is a common modality used in treating breast cancer and often presents a series of post-operative challenges. Seroma formation is the most common complication with no single method shown to be reliably effective against it. Fibrin glue is one such modality. There is a paucity of studies describing the utility of fibrin glue to prevent seroma formation.

Aim

To evaluate the effect of intraoperative application of fibrin glue sealant on drain output and seroma formation in patients undergoing modified radical mastectomy by comparing outcomes between the two groups.

Method

This is a prospective observational study. Fibrin glue application was done intra-operatively prior to closure in one group. Standard closure procedures with drain placement were undertaken in another group and their outcomes were analyzed for both groups.

Results

Drain output on postoperative day 1 (POD 1) as well as total drain output were recorded for both groups and the difference in mean was found to be significant (p<<0.05 and 0.049, respectively. Distribution of patients according to the volume of seroma formation was done; this difference of means was also found to be significant (p=0.004). Further, the difference of mean for the number of days the drain was in-situ was found to be significant.

Conclusion

Fibrin sealant used during modified radical mastectomy closures decreased the rates and volumes of seroma formation. This led to quicker drain removals and less patient discomfort.

## Introduction

Seroma formation is the most common postoperative morbidity experienced after modified radical mastectomies and no single method has been shown to be reliably effective against it [1]. Breast cancer has ranked as the number one cancer among Indian females [2]. Advances in surgery and oncology have now made breast cancer highly treatable [2,3]. Modified radical mastectomy with or without radiotherapy is indispensable to breast cancer treatment, and studies have shown the overall least rates of recurrence and systemic recurrence [4]. Post-operative complications of modified radical mastectomy include seroma formation, hematoma, loco-regional recurrence, nerve injury, dog ear deformity, skin flap necrosis, swelling of the arm due to lymphedema, myalgia, hemothorax, surgical site infection, etc. Seroma formation is the most frequent postoperative complication seen after mastectomy [5,6].

Seroma accumulation elevates the flaps from the chest wall and axilla thereby hampering their adherence to the tissue bed. It can lead to significant morbidity with complications including flap necrosis. It has also traditionally been assumed that the fluid collection is due to the drainage of lymph from the divided mammary and axillary lymphatics. On reviewing the literature, however, this appears to be more an assumption than a proven fact [1,4,7].

The pathogenesis of seroma has not been fully elucidated. The current understanding states that seroma formation develops due to acute inflammatory reaction to trauma and wound healing, which leads to increased fibrinolytic activity in serum and therefore increased serous fluid collection. Another school of thought believes that it is due to the severed lymphatics during surgery [8]. The incidence of seroma has been shown to correlate with the breast size [1], patient’s age [7], hypertension [9] presence of malignant nodes in the axilla, number of malignant nodes, previous surgical biopsy, and use of heparin [10]. The risk factors of seroma formation have been poorly established due to the prompt need for treatment taking priority in the clinical setting. Thus, there has been a significant focus on intra-operative interventions, post-operative interventions, and the combination of intra-operative and post-operative measures

A systematic review encompassing all Level I and II studies that discussed strategies for the prevention of postoperative seroma deciphered the effective strategy for seroma prevention included the use of closed-suction drains, keeping the drains until their output volume was minimal, maintaining a high-pressure gradient in the drains, using sharp or ultrasonic dissection rather than cautery, ligating blood vessels with sutures or clips, using quilting or progressive tension sutures, using fibrin, thrombin, or talc, and immobilizing the surgical site postoperatively [11]. These principles are applicable to general seroma but can also be applied to mastectomy. The use of fibrin glue is an intra-operatively utilized modality. Fibrin glue combines fibrinogen and thrombin in the presence of factor XIII and calcium chloride and generates a ’fibrin clot”’, similar to natural clotting cascade which acts as both a hemostatic agent and as an adhesive, closing over any small vessels, including lymphatics, which are not feasible for surgical closure. There is a paucity of studies objectively studying the effectiveness of fibrin glue in reducing post-operative seroma formation. This study aims to objectively study its effects on outcomes especially drain output and seroma formation.

## Materials and methods

The study was conducted in the department of General Surgery, Vardhman Mahavir Medical College and Safdarjung Hospital, New Delhi with the approval of the institutional ethics committee (IEC) and review board committee with approval no. of IEC/VMMC/SJH/October/2018-175.

This study aimed to evaluate the effect of intraoperative application of fibrin glue sealant on drain output and seroma formation in patients undergoing modified radical mastectomy by comparing outcomes between the two groups.

Method

A prospective observational study was conducted. Fibrin glue application was done intra-operatively before closure in one group. Standard closure procedures with drain placement were undertaken in another group and their outcomes were analyzed for both groups.

All patients presenting with breast nodules underwent investigations like fine needle aspiration cytology or a trucut biopsy to confirm carcinoma breast and a metastatic workup was performed for staging and treatment. Patients were included according to the criteria mentioned below (Table 1).

**Table 1:** Inclusion and exclusion criteria.

INCLUSION CRITERIA	EXCLUSION CRITERIA
Patients diagnosed with breast cancer posted for modified radical mastectomy.	History of axillary or radical thoracic surgery.
	Systemic anticoagulation or significant coagulation disorder.
	Platelet count ≤100,000/ml.
	History of chest radiation.
	Receiving preoperative chemotherapy.
	Planned immediate breast reconstruction.
	Pregnant or lactating women.

In a study by Docimo et al in patients undergoing mastectomy for primary breast carcinoma, seroma magnitude and duration were significantly reduced (p 0.004 and 0.02, respectively) [12]. Taking these values as a reference, the minimum required sample size with 90% power of the study and 5% of significance was 30 patients in each study group. Hence, the total sample size taken was 60 (30 patients per group). After their evaluation, patients classified as having breast carcinoma and not falling in the exclusion criteria were enrolled in the study with proper written informed consent. Patients were divided into two study groups, each having 30 patients as follows: -

· Group Standard closure (group 1): Patients undergoing standard closure techniques with drain placement only.

· Group Fibrin glue (group 2): Patients receiving Fibrin glue before standard wound closure and drain placement.

An institutional ethical clearance certificate was obtained before the commencement of the study. Data regarding age, comorbidities (diabetes, chronic liver disease, hypertension), serum albumin level, and breast mass size were recorded again one day before surgery. All patients underwent modified radical mastectomy by the same surgical team minimizing the use of electro-cautery as much as possible in both groups. The sequence of events intraoperatively was carried out as per the steps of modified radical mastectomy. Before carrying out flap closure, fresh fibrin sealant was prepared at the operating table and sprayed over the raw area of the chest wall along with the axilla and underneath the skin flaps.

Glue with the brand name “TISSEL LYO” (a two-component fibrin sealant with dulpoject injection system) manufactured by Baxter AG, A-1220 industriestrasse, BP-4-115 was used. This contained 6000 KIU synthetic Aprotonin and 1000 I.U human thrombin along with 144-250mg of human fibrinogen and other binding agents and sealer protein which makes the total protein 192-250mg. The fibrin sealant was prepared as follows: A vial of thrombin and a vial of fibrinogen was allowed to reach room temperature, after which the metal tabs and rubber bungs from both vials were removed, the container of sterile water for injection was opened and aspirated, and 2 mL was injected into the thrombin vial and 2 mL in fibrinogen vial (2mL in each). The vials were gently swirled for 1 min and then stood at room temperature for 5 minutes to ensure complete protein rehydration. Syringes were then placed in the duploject injector designed so that the common plunger exerted simultaneous and equal pressure on both the syringes. A mixer nose cone and topped blunt applicator needle were attached and the mixture was sprayed after completely drying the field. After spraying the mixture, gentle pressure was exerted for not less than 5 minutes over the flaps and axilla, and the surgical wound was closed as rapidly as possible after negative suction corrugated drains were placed in the axillary bed and underneath the mastectomy flaps and was clamped. Lastly, a small compressing cotton pad was placed in the axilla. Drains were unclamped after shifting the patient to the recovery room. Routine data collection was done every 12 hours and Performa was updated accordingly with attention to postoperative infection, amount of drained fluid on the first postoperative day, hospital stay, duration of drain in-situ, and amount and duration of postoperative seroma. Lastly, the number of excised lymph nodes and pathological results were also recorded. A seroma was defined as a clinically identifiable collection of serous fluid within a surgical cavity confirmed by more than 5 mL aspiration.

## Results

In this study, the mean age of all patients was 56.18 ± 12.77 years. The mean age of the patients in the standard group was 53.03 ± 11.68 and in the fibrin glue group was 59.33 ± 13.22. On examination, the majority of patients in both groups had nodules/lesions in the right breast. Among standard group patients, one-fifth (20%) of them had lesions on the upper outer surface of the breast, and among the fibrin group patients, more than one-fourth (26.66%) of the patients had lesions on the outer surface of the breast. On assessment for the size of the nodule, the mean nodule size was 8.17 ± 3.82 sq cm (skewness 0.510) for all patients. The mean size was 8.52 ± 4.1 sq cm and 7.82 ± 3.55 sq cm for the standard group and fibrin group repectively. Almost three fourth (70% of patients in group 1 and more than half of patients (56.66%) in group 2 had lymph nodes dissected from the level 3 group of lymph nodes. This distribution of proportions was found to be non-significant (p=0.284) (Table 2).

**Table 2:** Table showing preoperative factors of the standard group and the fibrin glue group.

Preoperative variables	Standard group (n=30) (Group 1)	Fibrin glue group (n=30) (Group 2)
Mean age of the patients	53.03 ± 11.68	59.33 ± 13.22
Mean nodule size	8.52 ± 4.1 sq cm	7.82 ± 3.55 sq cm
Percentage of patients with level 3 axillary nodes removal	70%	56.66%

Post-operative period

When observed for complications in the postoperative period, seroma formation was found to be significantly associated with a study group, with 70% of standard group patients presenting with it (p=0.037). A total of 73.3% of the standard group patients also required aspiration compared to 60% of the fibrin group patients. However, this difference in proportion was not found to be significantly (p=0.273). The majority of the patients (66.75% in group 1 and 53.35% in group 2) presented with skin flap necrosis, the distribution of which was not found to be significant (p=0.292). The mean drain output on POD 1 (mL) of the standard group was 144 ± 84.63, while that of the fibrin glue group was 80.33 ± 36.9. The mean total drain output (mL) of the standard group was 440.66 ± 213.78, while that of the fibrin group was 348 ± 132.06. The difference in comparison of means of drain output on POD 1 and total drain output was significant with the p-values being <<0.05 and 0.049 respectively. When assessed for the number of days drain in situ, it was observed that the mean days of the drain in situ for fibrin group patients were 4.3 ± 0.952 as compared to 5.9 ± 1.29 for patients of the standard group. This difference in mean days of the drain in situ for both groups was found to be highly significant (p<<0.05). When the distribution of patients according to the volume of seroma formation was done, it was observed that the difference of means among patients of both groups was found to be significant for the volume of seroma formation (p=0.004) (Table 3).

**Table 3:** Table showing the postoperative factors of both groups and their significance.

Postoperative variables	Standard group (n=30) (Group 1)	Fibrin glue group (n=30) (Group 2)	P-value
Seroma formation	21	13	0.037
Skin flap necrosis	20	16	0.292
Mean drain output on POD 1 (mL)	144 ± 84.63	80.33 ± 36.9	<<0.05
Mean total drain output(mL)	440.66 ± 213.78	348 ± 132.06	0.049
Volume of seroma (mL)	306.67 ± 430.67	59 ± 124.21	0.004
Mean of No. of days with the drain in situ	4.3 ± 1	5.9 ± 1.29	<<0.05

**Figure 1:**
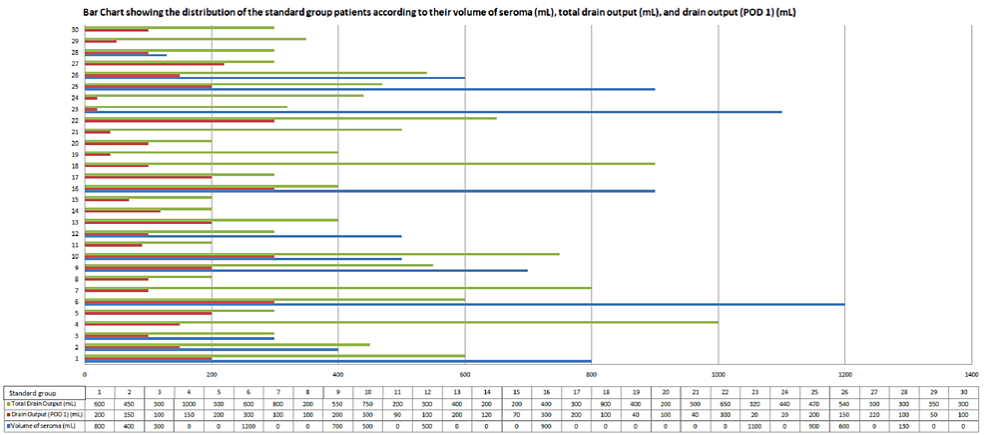
Bar chart showing the distribution of the standard group patients according to their volume of seroma (mL), total drain output (mL), and drain output (POD 1) (mL).

The following data is the representation of the distribution of the standard group patients according to their volume of seroma (mL), total drain output (mL), and drain output (POD 1) (mL) (Figure 1).

**Figure 2:**
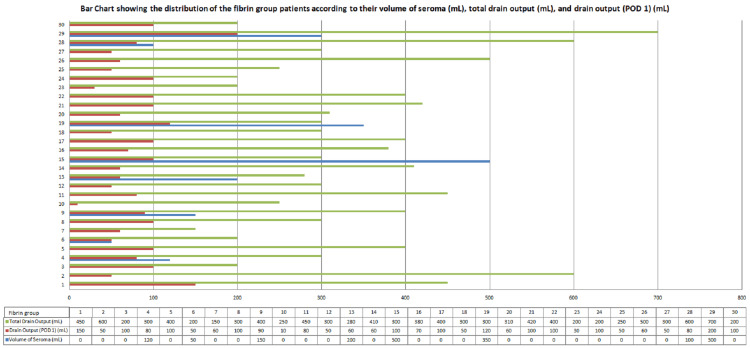
Bar chart showing the distribution of the fibrin glue group patients according to their volume of seroma (mL), total drain output (mL), and drain output (POD 1) (mL).

The following data is the representation of the distribution of the fibrin glue group patients according to their volume of seroma (mL), total drain output (mL), and drain output (POD 1) (mL) (Figure 2).

## Discussion

Breast cancer is the leading cancer among women in India. Modified radical mastectomy is the most important surgical treatment modality for the same. Seroma formation is the most common complication occurring post-operatively in patients undergoing modified radical mastectomy. This causes an undue increase in the morbidity of the procedure and eclipses patient care standard and outcomes. It is frequently considered a cause of complications like skin flap necrosis as well. In this study, patients were found to be mostly in the age group of 40-60 years with the majority of patients in both groups having nodules/lesions in the right breast on the outer surface with a mean size of 8.17±3.82 sq cm. Both the groups underwent modified radical mastectomy by the same surgical team, and almost three fourth of the patients had lymph nodes dissected from the level 3 group of lymph nodes. Even though both groups experienced complications, seroma formation was found mostly in the standard group. Fibrin glue application resulted in significantly lower rates of seroma formation, volume of seroma formation, and drain output on postoperative day 1, the results of which are comparable to a study conducted by Van Bastelaar et al [13]. Patients in the fibrin group had to undergo a smaller number of drainage procedures for seroma as compared to a standard group, which was consistent with the studies done by Docimo et al [12]. This can be attributed to the action of fibrin on severed lymphatics and obliteration of dead space using adhesion. Thus, seroma formation is reduced in the postoperative period, while delayed seroma formation and seroma volumes may not be affected by it due to the overlap of lymphorrea and inflammatory pathways. There was also a significant reduction in the total number of days for which the drain was in-situ, which was consistent with the results of a study conducted by Fawzy et al [14] which assisted in better managing seroma at the least, can significantly reduce hospital stay along other drain related complications, and reduce the burden on health care facilities.

A major limitation is a missing follow-up, lack of info on the duration of seroma control, subjective decision of need for aspiration of seroma, and potential complications, immediate or delayed. This study was conducted in a single tertiary care center with a limited study population. multicentric study with a larger study population will be required to establish the correlation between the fibrin glue sealant use and seroma formation.

## Conclusions

Fibrin glue sealant is a useful adjunct to decrease seroma formation and skin flap necrosis in patients undergoing MRM. It lowers the drain fluid amounts leading to quicker drain removal, lesser morbidity, and improved comfort.
